# Stigma and Peer-Led Interventions: A Systematic Review and Meta-Analysis

**DOI:** 10.3389/fpsyt.2022.915617

**Published:** 2022-07-05

**Authors:** Jing Sun, Xunbao Yin, Changjiang Li, Wuyi Liu, Hongwei Sun

**Affiliations:** ^1^School of Public Health, Weifang Medical University, Weifang, China; ^2^School of Teacher Education, Weifang University, Weifang, China; ^3^School of Psychology, Weifang Medical University, Weifang, China

**Keywords:** stigma, peer-led intervention, meta-analysis, HOP intervention, systematic review

## Abstract

**Purpose:**

The main purpose of our systematic review was to investigate the effect of peer-led intervention on self-stigma in individuals with mental health problems. Secondary purpose was investigating the impact of peer intervention on clinical symptoms, recovery-related outcomes, and disclosure-related outcomes.

**Methods:**

Five electronic databases were searched from 1975 to 2021. Literature databases were searched for randomized controlled trials. From the perspective of key outcomes, a meta-analysis of the effects of peer-led interventions on changing stigma was conducted.

**Results:**

A meta-analysis of randomized controlled trials targeting different target groups with mental health problems (e.g., adolescents, college students, family members of mentally ill persons, unemployed persons, etc.) was conducted. It was found that, at the end of the intervention, intervention had a positive effect on main outcomes such as self-stigma and stress from stigma. As for secondary outcomes, there was no significant influence on clinical symptoms. There was a positive effect on rehabilitation and empowerment, but without a statistical significance. There was a statistically significant effect on self-efficacy and professional help seeking. There was a statistically significant effect on confidentiality and disclosure-related distress in the Honest Open Proud (HOP) subgroup. There was no significant influence on confidentiality and withdrawal in the non-HOP subgroup.

**Conclusion:**

Peer-led intervention can reduce self-stigma and stigma pressure and might improve recovery and empowerment. It increases self-efficacy and willingness to seek professional help, but has no significant effect on clinical symptoms and withdrawal. HOP intervention has positive effects on disclosure-related confidentiality and pain.

**Systematic Review Registration:**

https://www.crd.york.ac.uk/PROSPERO, identifier: CRD42021287584.

## Introduction

Stigma has long been recognized as a major challenge in the treatment and recovery of mental illness (1). Stigma includes three aspects: cognition (stereotype), emotion (prejudice), and behavior (discrimination) (2). Individuals with mental illness often encounter two stigma-related challenges: public stigma, and self-stigma (3). On the one hand, they may experience public prejudice and discrimination, thus using secrecy or withdrawal strategies to cope with this situation (4). On the other hand, they are not only aware of negative public stereotypes about them, but also stigmatize themselves when they identify with these stereotypes. Namely, self-stigma is self-devaluation after accepting and internalizing the related negative perceptions and stereotypes (1). People with mental illness are more likely to internalize stigmatizing belief due to social exclusion, discrimination, isolation, and more misunderstandings from the society (5). Studies have shown that the proportions of the psychiatric patients with self-stigma are 41.7% (6) and 36.1% (7) in Europe and the United States, respectively. A review by Livingston and Boyd (8) demonstrated that high level of self-stigma were significantly associated with feelings of hopelessness, poorer self-esteem, decreased empowerment/control ability, and decreased self-efficacy, which contributed to increased psychiatric symptoms and suicidal ideation, lower self-esteem, social withdrawal, limited social support, and low willingness to seek professional help (9–11). It was found that self-stigma had a remarkable negative impact on individual health and social function, and might increase social burden (12). At the macro level, it is of great necessity to add approaches of reducing public stigma of mental illness into mental health policies (13), as these approaches can alleviate stereotype identification. However, groups of different racial and cultural backgrounds may need to face other challenges such as racial discrimination (14) and the effects of these approaches were not satisfactory due to high costs and poor precision. Nonetheless, micro-interventions targeting self-stigmatization of individuals with mental illness may be a promising way.

In recent years, the interventions aiming at reducing self-stigma can be classified into the following types: psychoeducational intervention, cognitive-behavioral intervention, disclosure-focused intervention, and combined interventions. Psychoeducational intervention can help the patients gain more knowledge of mental illness and enhance the understanding and ability to deal with the stigma of mental illness (15, 16). Psychoeducational intervention mainly trains the patients in critical thinking on the knowledge about mental illness but does not reduce stigma perception and internalization (17). Studies have also confirmed that only using psychoeducational approach was limited in improving self-stigma (15). Cognitive-behavioral therapy decreases self-stigma by correcting distorted self-concepts (18), improving low self-esteem, and reducing avoidance behaviors (19, 20). The effect of cognitive-behavioral therapy on self-stigma intervention is limited but beneficial (18), and it also improves recovery and depression (21). Corrigan et al. (22) proposed a disclosure-focused approach, named as Honest, Open, Proud (HOP, formerly called Coming Out Proud, COP), which focused on discussing the pros and cons of disclosure or confidentiality in different contexts. Contact is key part in disclosure. When people with mental illness interact with their convalescent peers, strategic disclosure can promote peer relationships, reduce self-stigma (23), and exerts positive effects on depression, recovery, and quality of life (24).

One of the key parts in the development of mental health services is the transition to the recovery model which focuses on the subjective sense of wellbeing of the service users (25). Recovery is significantly negatively correlated with self-stigma (12). Peer support is a recovery-oriented intervention (26). Solomon (27) believed that peer support was provided by individuals with similar mental health conditions either mutually or unidirectionally to generate social and emotional support which results in expected social or personal changes. One of the core processes of peer support is social support (28), and one of the key principles is enhancing empowerment (29). Research showed that the rehabilitation-oriented mental health interventions provided by the cooperation of professionals and peer providers could promote recovery, enhance empowerment, and increase hope (30). However, the effect of peer intervention on self-stigma remains to be studied. This systematic review and meta-analysis aims to integrate evidence from randomized trials to illustrate the effects of peer-led group intervention on self-stigmatization and its related problems in individuals with mental health problems.

## Methods

The research methods of this review were conducted in accordance with the Cochrane Collaboration Guidelines for systematic reviews of interventions and reported in accordance with the Preferred Reporting Items for Systematic Reviews and Meta-Analysis (PRISMA) statement (31). Before conducting this review, this study was registered on PROSPERO (32).

### Search Strategy, Study Selection, and Data Extraction

#### Search Strategy

Data was searched from the following 6 databases: Embase, Pubmed, APA PsycInfo, APA Psyc Tests, Cochrane and Clinicaltrial. Language was not restricted in literature search. Non-English papers have been screened out since they do not meet the standards of PICO, and the final selected studies are published in English. The following search strategy was used. Search terms were (“peer group” or “peer-led”) and (“stigma”). Details on the search of MeSH terms and the cognates were illustrated in Annex 1. The titles and abstracts of the search results were first independently screened by two PhD students. After the initial screening, the full text was read to determine whether the article could be included. If there is any doubt on the accepted literature, authoritative tutors would make a decision on the inclusion of the paper. The tutors were provided with the screening instructions in advance.

#### Inclusion and Exclusion Criteria

We only included randomized controlled trials following a completely randomized design. Published, unpublished, and completed trials were eligible for inclusion. Cluster RCTs, incomplete RCTs, and all the studies of non-randomized designs, including partially randomized and quasi-experimental designs, were excluded. Papers were included if they reported the following aspects: any design assessing the effects exposing any type of peer-led intervention to the participant; participants were those who had any mental health problem or experience mental health issue; the aim of the intervention was to reduce the stigma of mental illness. Papers were excluded if they reported the following aspects: peer-led interventions were not conducted face-to-face; peer-led interventions were informal; studies were case reports or not related to the stigma of mental illness. The study were also excluded if the study only included participants who had organic neuropathy (e.g., dementia), a disease usually diagnosed during childhood (e.g., conduct disorder), developmental disorder (e.g., autism), or alcohol- or drug-abuse-related disorders.

#### Data Extraction

Data were extracted from eligible original papers, including study design, description of the intervention, inclusion criteria and demographic characteristics of participants, statistical data on outcomes (sample size, mean, and standard deviation) at the end of the intervention. The primary outcomes were self-stigma and stigma stress. Secondary outcomes were clinical symptoms, such as depression, anxiety, and hopelessness; recovery-related factors, such as recovery, empowerment, self-efficacy, and help-seeking; disclosure-related factors, such as confidentiality, disclosure-related distress, and withdrawal.

### Statistical Analysis

Considering that the sample sizes of relevant studies were often too small to ignore the effect of baseline bias, we integrated and analyzed the data according to the principle of “change from baseline.” All the extracted data were continuous variables, and thus the standardized mean difference (SMD) with 95% confidence interval was used. Pooled effect sizes were calculated by fixed-effects (*I*^2^ <50%) or random-effects (*I*^2^ ≥ 50%) model. Even if our study collected the data on follow-up results, we only analyzed the outcomes at the end of the intervention (the records at the end of the treatment).

All statistical analyses were performed using Revman 5.4. Heterogeneity was investigated by using the sensitivity analysis of “removing one study.” In the sensitivity analysis of “removing one study,” a single study was removed one by one each time to determine the effect of this study on the observed effect. The number of the studies reporting the indicators included in this review was <10, and thus we did not conduct publication bias analysis. If the data of certain results could not be extracted, a narrative description of the results was created.

## Results

**Figure 1:**
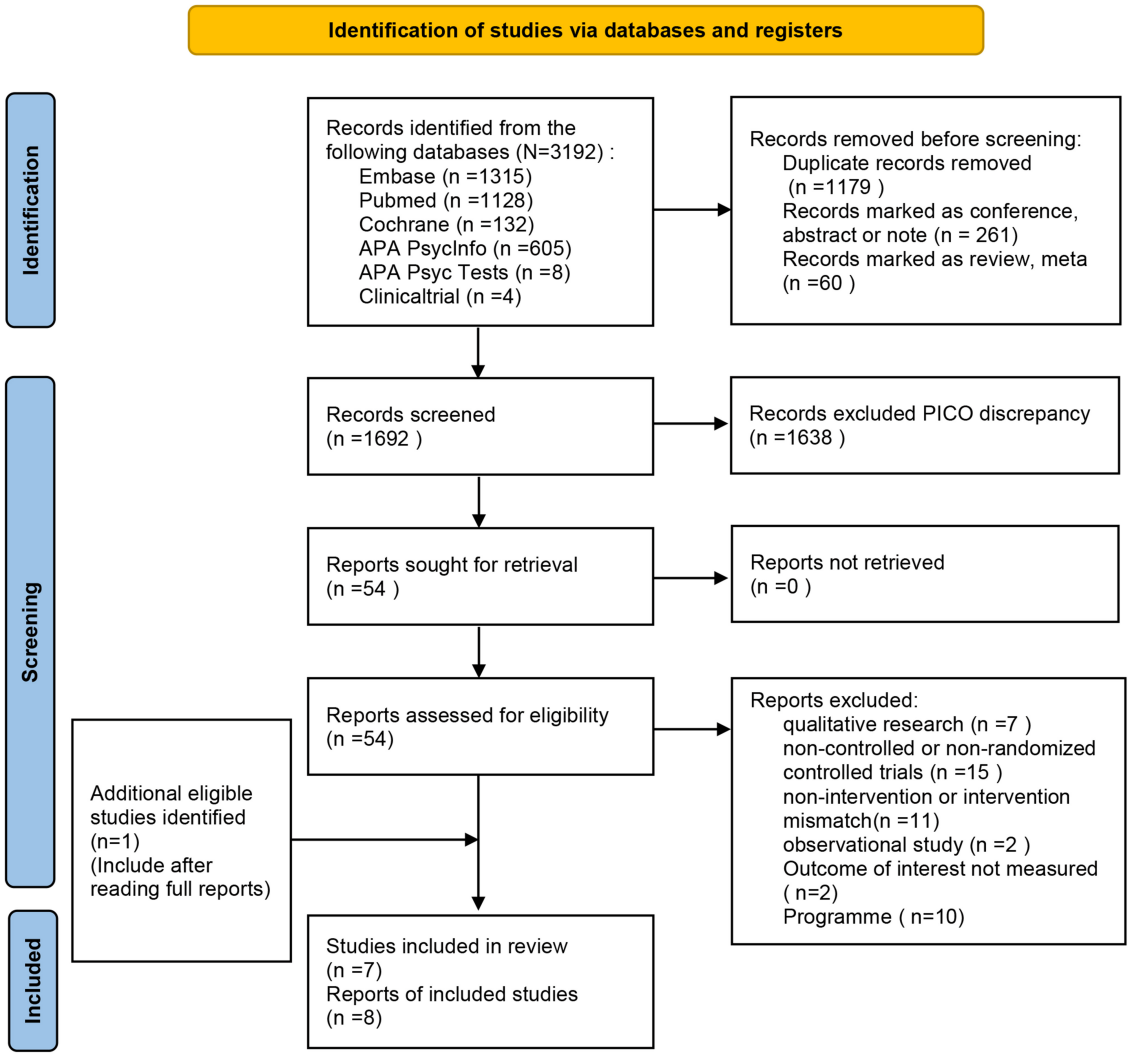
PRISMA flow chart.

**Figure 2:**
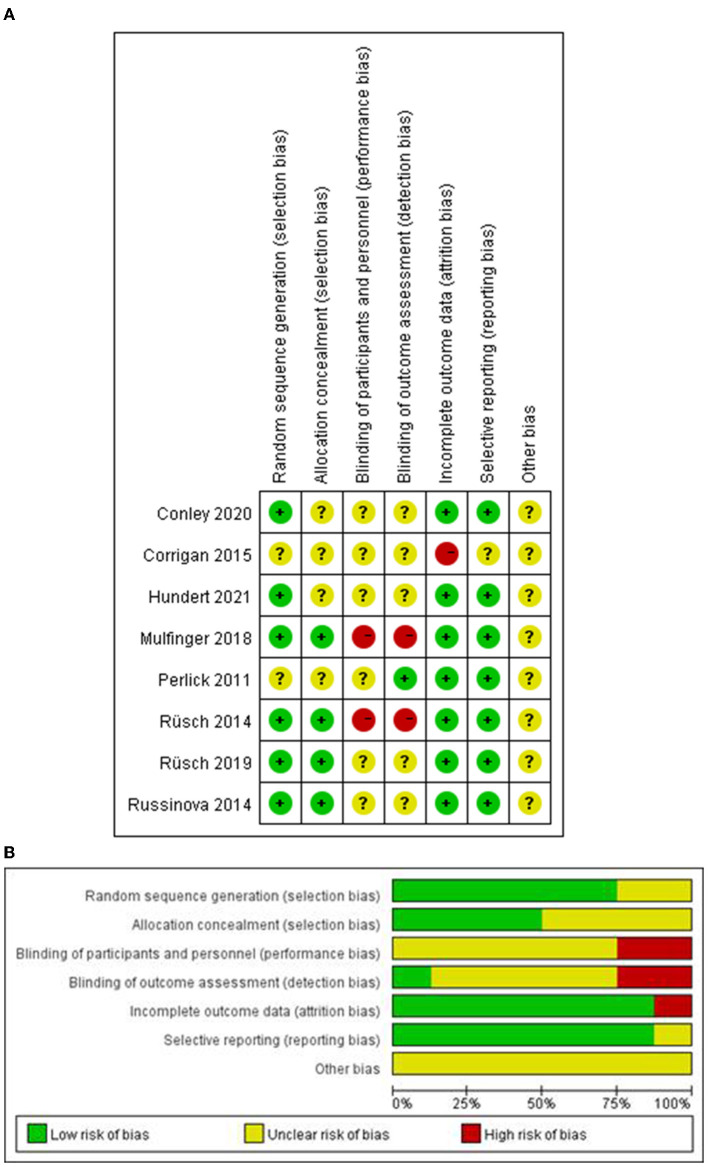
Risk of bias assessment.

A total of 3,192 records were retrieved after searching the database. Titles and abstracts of 1,638 records were screened for eligibility after removing the duplicates, conference abstracts and reviews. We excluded 1,692 articles and recorded clear exclusion criteria: the title or abstract was irrelevant of our study type, intervention type or study population. A total of 54 articles were searched for full text, among which 6 studies and 7 articles were included. When we read the full text, we found that one article that met the inclusion criteria was not retrieved, and thus 8 articles were included (the complete PRISMA flow chart was shown in Figure 1). The assessment of ROB was shown in Figure 2, indicating that these studies were generally of low/moderate risk. As data from one paper could not be extracted, useful data from seven reports were applied in this meta-analysis. The meta-analysis on the data included stigma (self-stigma and stigma stress), clinical symptoms (depression, anxiety, and hopelessness), personal recovery (self-efficacy, empowerment recovery, and seeking help), and disclosure-related outcomes (confidentiality, distress, back off). Sensitivity analyses of the results showed that the heterogeneity of most outcomes did not change the results, except for self-efficacy, empowerment, confidentiality, and help-seeking. Since the heterogeneity of the confidential results had a great impact on the results, two subtypes were created according to the characteristics of the intervention: HOP interventions and non-HOP interventions.

### Characteristics of the Literature

All the included reports were randomized controlled trials using the parallel group design. Five studies (33–37) were conducted in the United States, two studies (38, 39) in Germany and one study (40) in Switzerland. Five studies (34, 35, 38–40) reported the follow-up data of 3 weeks to 6 months after treatment. Details were shown in Table 1.

**Table 1:** Study characteristics.

**Study ID**	**Intervention and (control) groups**	**n (base line)**	**Diagnoses**	**Sex %F**	**Ethnicity %BAME**	**Age**	**Key findings as reported in paper**
Conley et al. (33)	HOP-C(WLC)	118	• 85.5% CES-D>10 • 69.2% GAD-7>10	82.2	44.2	20.8	• Self-stigma (SSMIS-SF) • Stigma appraisals • Self-efficacy (disclosure or secrecy) • Mental health symptoms (CES-D, GAD-7)
Corrigan et al. (34)	COP(WLC)	126	N/R	63.5	64.5	45.6	Self-stigma (SSMIS) Stigma stress Depressive symptoms (CES-D)
Hundert et al. (35)	HOP-C(WLC)	75	85.45% CES-D>10 67.27% GAD-7>10	85.5	N/R	19.2	Self-Stigma (SSMIS-SF) Stigma appraisals Self-efficacy (disclosure or secrecy) Mental health symptoms (CES-D, GAD-7)
Mulfinger et al. (38)	HOP+TAU(TAU)	98	64% (58%) DD 19% (17%) AD	69.4	N/R	15.8	Stigma stress Quality of life Self-stigma (ISMI SSMIS) Empowerment Disclosure-related Help-seeking Hopelessness Attitudes to disclosure
Perlick et al. (36)	IOOV-FC(FE)	122	100% CSS	73.4	17.2	56.7	Self-stigma Secret Withdrawal
Rüsch et al. (40)	COP+TAU(TAU)	100	56% (64%) DD 18% (22%) BPD 16% (11%) SS	59.0	2.0	42.0	Self-stigma Empowerment Stigma stress Disclosure-related Secret Perceived benefits of disclosure
Rüsch et al. (39)	HOP+ACT(TAU)	42	100% KKPDS≥13 100% MD≥3	52.0	N/R	46.1	Job-search self-efficacy Help-seeking intentions Recovery (SISR) Self-stigma (SSMIS-SF) Depressive symptoms (CES-D) Hopelessness (BHS) Secret
Russinova et al. (37)	PV(TAU)	82	34% SS 33% BPD 26% DD 7% other	68.0	31.0	68% were > 40 years	Self-stigma (ISMIS) Coping with stigma Recovery (PGRS) Empowerment Depressive symptoms (CES-D) Self-efficacy

*AD, anxiety disorder; BHS, beck's hopelessness scale; BPD, bipolar disorder; CES-D, center for epidemiologic studies- depression scale; CSS, caregivers of schizophrenia spectrum disorder; DD, depressive symptoms; FE, family education; GAD-7, generalized anxiety disorder 7-item scale; HOP-C, honest open proud-college; IOOV-FC, in our own voice– family companion; ISMIS, internalized stigma of mental illness scale; KKPDS, kessler's k6 psychological distress scale; MD, mental distress; N/R, not recorded; PGRS, Personal Growth and Recovery Scale; PV, photovoice; WLC, waiting list control; SISR, self-identified stage of recovery scale; SS, schizophrenia spectrum disorder; SSMIS-SF, self-stigma of mental illness scale-short form; TAU, treatment as usual*.

### Characteristics of the Subjects

The total number of the included subjects in the study was 763, including 373 in the experimental group and 389 in the control group. The median study size was 99 with a range of 42–126 participants. The experimental group included the population at the following ages: adolescent, college student, middle-aged and elderly (mean age >40) participants. The proportion of median number of female participants was 68.7%, ranging from 52.0 to 85.5%. The proportion of Black, Asian and minority participants ranged from 2 to 64.5%. The eligibility criteria for the participant in most trials included a series of mental health diagnoses. One was a study focusing on the caregivers of serious mental illness (36); One study did not report the diagnoses of the participants, but they all used mental health services (34).

### Characteristics of Intervention

The included studies were all randomized studies using peer-led intervention. The intervention lasted 3–10 weeks and employed structured group interventions provided by 1–2 peer counselors to reduce self-stigmatizing behaviors and improve their responses to experienced stigma. HOP intervention was used in 5 groups. HOP is a peer-led group intervention developed by Corrigan et al. (41), formerly known as “Coming Out Proud (COP).” HOP intervention aimed to reduce self-stigma and its effects in those individuals with mental illness by empowering participants to disclose their mental health status in different conditions. The purpose is not encouraging disclosure, but to disclose their information with caution. The decision of disclosure is individual and dependent on surrounding environment. HOP classes were led by trained individuals with mental illness, which included vignettes, role-plays, self-reflection exercises, and group discussions on disclosure.

Mulfinger et al. (38) investigated whether a 3-session HOP program had a positive effect on stigma stress and quality of life among adolescents (mainly hospitalized). Both Corrigan et al. (34) and Rüsch et al. (40) adopted the “Coming Out Proud (COP)” program to disclose mental illness. Rüsch investigated the effect of 3-session COP program on reducing stigma and promoting adaptive coping skills. Corrigan offered a 2-day course to explore the impact of COP on stigma and stigma stress and examined its clinical significance. Conley et al. (33) and Hundert et al. (35) studied the impact of HOP-C on stigma of mental illness among college students and how to disclose mental status. HOP-C was revised from HOP which included information more relevant to college students. Rüsch et al. (39) used the combined intervention of ACT and HOP in unemployed people with mental health issues to support their disclosure decisions.

Russinova et al. (37) used the intervention method of Photovoice, a participatory research method (42), in which participants used a camera to record their life and developed a narrative on the meaningful personal visual images (43). This method was used as a medium for disease communication and advocacy (44). Russinova et al. applied this method as an anti-stigma intervention and piloted a 10-week anti-stigma Photovoice intervention by peer-led through exercise books and leadership guides. It was a program combining psychoeducation and experiential exercises, which used photography and storytelling to tackle stigma.

Perlick et al. (36) adopted the IOOV intervention method. IOOV, “In Our Own Voices, Living with Mental Illness,” is an anti-stigma program proposed by the American Alliance for Mental Illness. The host and team members are the primary caregivers in the family of the mentally ill, they first watched a video on coping with mental illness stigma based on the framework of Stages of Emotional Response and then the moderator leads the members have a discussion. This program combined contact and education and included the following sections: dark days, acceptance, healing, coping and success/hope/dream. Via interventions, the public could know about mental illness, change their attitudes toward mental illness, and reduce stigma against mental health consumers (45). Perlick conducted a 3-stage IOOV-FC intervention, by contacting and comparing the views and coping strategies of others, to change self-awareness, promote family peer interaction, and break down the negative and internal stereotypes of family members.

The control group received conventional treatment (37–40) or home education (36), or they were on the waiting list (33–35).

### Main Results

**Figure 3:**
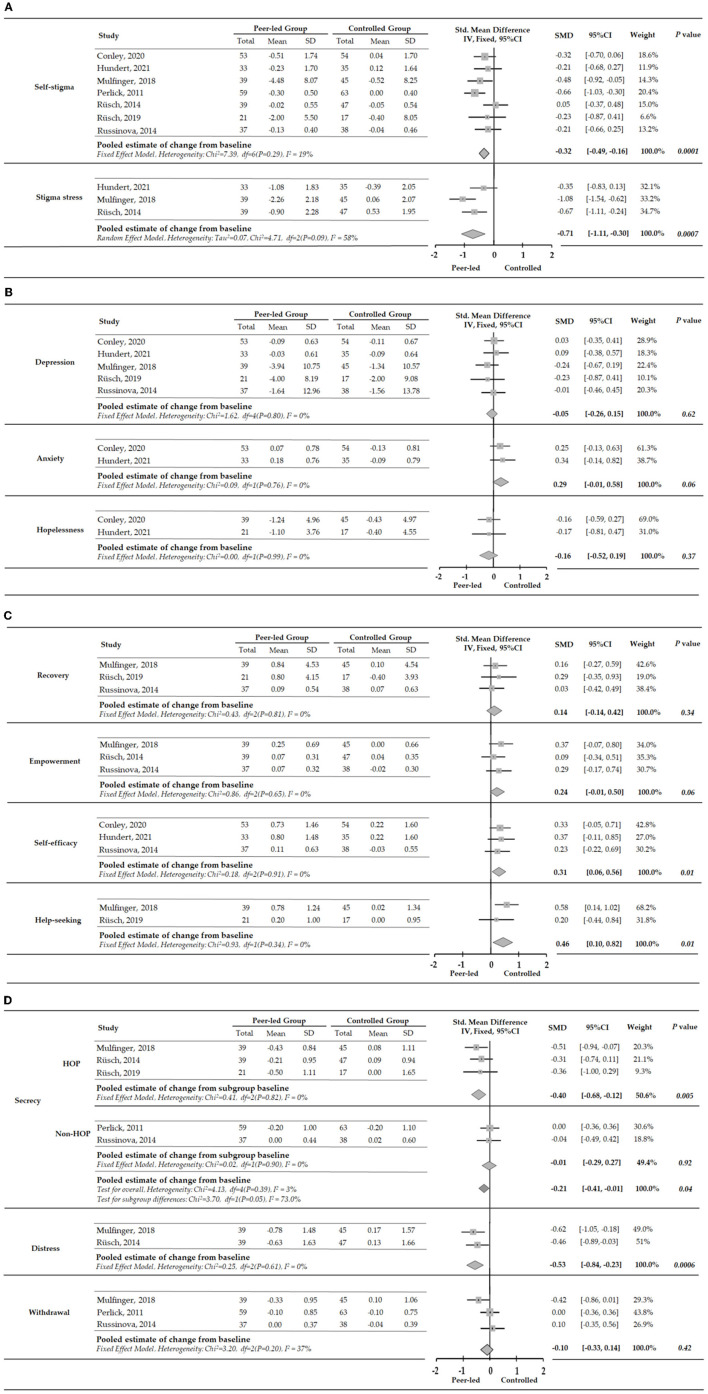
**(A)** Meta-analysis of peer-led effects on stigma at the end of intervention. A negative SMD (to the left) indicates a positive effect. **(B)** Meta-analysis of peer-led effects on clinical symptoms at the end of intervention. A negative SMD (to the left) indicates a positive effect. **(C)** Meta-analysis of peer-led effects on individual rehabilitation outcomes at the end of intervention. **(D)** Meta-analysis of peer-led effects on disclosure-related results at the end of intervention. A negative SMD (to the left) indicates a positive effect.

The meta-analysis on the integrated results showed that peer intervention had a statistically significant effect on self-stigma and stigma stress. Self-stigma (*I*^2^ = 19%, fixed effects model, SMD = −0.32, 95%CI [−0.49, −0.16], *P* = 0.0001) and stigma pressure (*I*^2^ = 58%, random effects model, SMD = −0.71, 95%CI [−1.11, −0.30], *P* = 0.0007) were significantly reduced (Figure 3A). Corrigan et al. (34) reported that the harm in self-stigma [*F*_(1, 44)_ = 6.49, *P* <0.01], application of stereotype [*F*_(1, 44)_ = 6.67, *P* <0.05), stigma protocol [*F*_(1, 43)_ = 6.04, *P* <0.05] and stigma harm in stigma stress [*F*_(1, 43)_ = 8.45, *P* <0.01], and coping resources [*F*_(1, 44)_ = 5.30, *P* <0.05] were significantly changed after the intervention. However, this study did not provide extractable data and therefore the results could not be analyzed quantitatively.

### Secondary Results

#### Clinical Symptoms

The results synthesized by meta-analysis showed that peer intervention had no statistically significant effect on depression (*I*^2^ = 0%, fixed-effects model, SMD = −0.05, 95%CI [−0.26 to 0.15], *P* = 0.34), anxiety (*I*^2^ = 0%, fixed-effects model, SMD = 0.29, 95%CI [−0.01 to 0.58], *P* = 0.06), and hopelessness (*I*^2^ = 0%, fixed effects model, SMD = −0.16, 95% CI [−0.52 to 0.19], *P* = 0.37) (Figure 3B). Corrigan et al. (34) reported that, compared with the control group, depression was significantly ameliorated in female group after peer support intervention [*F*_(1, 33)_ = 8.15, *P* <0.01], while no significant change was observed in male group. However, this study did not provide extractable data and therefore the results could not be analyzed quantitatively.

#### Individual Rehabilitation Outcomes

The results synthesized by meta-analysis showed that peer intervention had a statistically significant effect on self-efficacy (*I*^2^ = 0%, fixed-effects model, SMD = 0.31, 95% CI [0.06, −0.56], *P* = 0.01) and help-seeking (*I*^2^ = 0%, fixed-effects model, SMD = 0.46, 95%CI [0.10, −0.80], *P* = 0.01). The effect of peer intervention on empowerment (*I*^2^ = 0%, fixed effects model, SMD = 0.24, 95% CI [−0.01, −0.50], *P* = 0.034) and rehabilitation (*I*^2^ = 0%, fixed effects model, SMD = 0.14, 95% CI [−0.14, −0.42], *P* = 0.0 34) was not statistically significant (Figure 3C).

#### Disclosure-Related Results

The results synthesized by meta-analysis showed that peer intervention had a statistically significant effect on disclosure-related secrecy (*I*^2^ = 3%, fixed-effects model, SMD = −0.21, 95% CI [−0.41, −0.01]) and distress (*I*^2^ = 0%, fixed-effects model, SMD = −0.53, 95% CI [−0.84, −0.23], *P* = 0.0006). The intervention effect on withdrawal (*I*^2^ = 37%, fixed-effects model, SMD = −0.10, 95% CI [−0.33, 0.14], *P* = 0.42) was not statistically significant. Secrecy was significantly reduced in the HOP subgroup (*I*^2^ = 0%, fixed-effects model, SMD = −0.40, 95% CI [−0.68, −0.12], *P* = 0.005). The decrease in secrecy was not statistically significant in non-HOP subgroup (*I*^2^ = 0%, fixed-effects model, SMD = −0.01, 95% CI [−0.29, −0.27], *P* = 0.92), and the heterogeneity between the groups was high (*I*^2^ = 73%) (Figure 3D).

## Discussion

Through a comprehensive literature search and reading, 8 randomized controlled trials were identified. This study evaluated the effects of peer intervention on self-stigma, clinical symptoms, and recovery of mental illness. The interventions included in the studies were all one-way group services provided by trained peers. Most studies used structured interventions which tended to combine psycho-education, exercise books and group discussions, and fidelity test on the content of the interventions was conducted. There are three types of interventions: HOP, IOOV-FC, and PhotoVoice. Five of the 7 studies used HOP intervention and the other two used IOOV-FC and Photovoice, respectively.

This study showed that peer intervention could improve self-stigma and stigma stress. A comprehensive cognitive model of self-stigma demonstrated that peer support was one of the protective factors of self-stigma, and the key factor that played a protective role is group identity (18, 46). Research hypothesized that individuals with mental health problems could establish positive connections with their convalescent peers in interactions and reduce self-stigma (29). The results of this systematic review and meta-analysis confirmed this hypothesis, which was greatly consistent with the results found by Griffiths et al. The research believed that active contact with peers could reduce the stigma in the patients with mental illness. The mechanism of peer intervention in influencing self-stigma may lie in the following aspects. First, the key point of peer intervention is “homogeneity,” which makes team members more likely to establish connections (47). Second, “social support” is also a key factor. The understanding, empathy, and help provided by peers (48) can strengthen the bonds and encourage individuals to become members of the group. Finally, “identification” is another core factor. By sharing their own experiences or recovery stories, peer moderators may bring a closer relationship, reduce stereotypes, and forms a positive sense of identity and group identity, thereby reducing self-stigma. Stigma stress is proposed based on stress model, which is a key response of the individuals facing the threat of discrimination (49) and is associated with negative outcomes (50). The evaluation of stigma is one of the reasons that lead to self-stigma, during which stigma pressure (50) can also be generated. The evaluation of stigma pressure is influenced by the comment of the group by the members in the group (50). Peer-led intervention influences the three main factors affecting group evaluation through the impacts of role-model. The factors included promoting a positive sense of group value (perceived group value), strengthening the sense of identity in group members (group identification), and making the group members feel connected with a meaningful group (substantiality) and have more coping resources, thereby enhancing their resilience to stigma and reducing stigma stress assessment (51).

This study showed that there was no significant effect of peer intervention on clinical symptoms. According to the modified label theory, reduction in self-stigma may improve symptoms (52, 53). However, this study found that although self-stigma was reduced, the peer-led intervention had no significant effect on clinical symptoms. This might be resulted from the following reasons. First, although peer support provides positive feedback and increases available resources, improving symptoms is not the main effect of peer support (54). Secondly, recovery outcomes might be more appropriate than clinical outcomes in the evaluation of peer support (55). Studies have demonstrated only a mild to moderate correlation was found between clinical symptoms and personal recovery (56).

Peer-led interventions tended to improve recovery and empowerment, but the improvement was not statistically significant. Statistical significance was found in improving self-efficacy and help-seeking. Most of the research on coping with self-stigma included in this study was rehabilitation-oriented. Recovery is the process of overcoming the challenges of mental illness and gaining significance and sense of achievement (30). Self-stigma may be a major barrier to recovery (23). The results of this study showed that peer intervention had a tendency to improve rehabilitation but without statistical significance. This result was not consistent with the findings by Lyons et al. (57) who believed peer support had a significant positive impact on rehabilitation. Peer support could provide social support related to health and positive recovery outcomes (28). However, the measures of rehabilitation included in this study are inconsistent, Mulfinger et al. (38) and Rüsch et al. (39) measured rehabilitation on the Self-Identified Stage of Recovery Scale, and Russinova's Scale for Personal Growth and Recovery Scale (PGRS) developed by their team to assess participants' perceived recovery and growth. Given the different evaluation criteria for the included rehabilitation, or the confusion over the different infrastructures of rehabilitation, caution is needed to elaborate on which specific components of peer support can improve rehabilitation need further research. Empowerment is a process in which individuals can gain control over important issues in their lives (58), a continuum opposite to self-stigma (59), and one of the key parts in recovery (60). As mentioned previously, peer intervention promoted positive self-disclosure and the establishment of supportive resource pool, which not only enhanced the sense of engagement in the individual (engagement) but might also improve the individual's ability to mobilize resources (control). Since these two are the core aspects of empowerment (61), peer intervention may enhance empowerment. The results of this study showed that empowerment tended to be improved but without statistical significance. Similar as the results in rehabilitation, the included studies used inconsistent evaluation methods. Mulfinger used self-esteem and optimism to reflect the strength of empowerment. Rüsch et al. (40) and Russinova used 28-item empowerment scale. The scale consists of five dimensions (self-efficacy-self-esteem, power-powerlessness, community activism and autonomy, optimism and control over the future, and righteous anger). That is, the dimensions of measurement empowerment are not consistent. Self-efficacy belongs to empowerment (62) and is the individual's belief in the ability to achieve desired outcomes (63). Peer support could improve self-efficacy and this result was relatively consistent with other research (29). Individuals observe and interact with their convalescent peers and learn the experiences of successfully coping with disease from them, which exerts the effect of vicarious reinforcement. This may enhance the positive beliefs of being able to deal with their own disease and increase self-efficacy. Psychiatric patients with internalized stigma may avoid being rejected by keeping a distance from others and refusing to establish connection with others, and thus they are more likely to avoid seeking help (64, 65). The improvement effect of peer intervention on help-seeking may be originated from the “sense of normality” generated by “homogeneous.” The upward comparison with excellent “homogeneous” individuals brings hope and provides motivation for upward development (66), and thus they are more willingly to seek help. However, the complex mechanism and individual differences may lead to different effects.

The meta-analysis on disclosure-related outcomes showed that peer-led intervention had a positive effect on confidentiality in the HOP subgroup, but no improvement in the non-HOP subgroup. The intervention had a positive effect on disclosure-related distress, but no improvement in withdrawal. Research suggested that disclosure was the first step in empowerment (1). One of the approaches to fight against stigma is to disclose, which is letting others know about their mental illness. When being open and honest, individuals may worry less about secrecy, find supportive resources, enhance the sense of power and increase control over their lives (67). Participants in HOP intervention could learn new and diverse approaches to dealing with the complexities of disclosure, thereby reducing the impact of stigma as a stressor when they encountered with disclosure decisions. Meanwhile, disclosure-related distress could also be ameliorated. The design of the intervention applied in non-HOP subgroup did not focus on disclosure, but on learning, discussing and sharing stigma-related experiences and coping strategies among members with similarities. Thus, the intervention applied in non-HOP subgroup had no significant effect on confidentiality. The change in withdrawal was not significant, which might be due to the sample differences and the use of different strategies. Mulfinger' s research adopts HOP intervention, and the research group is mainly adolescents with attention deficit, behavior, anxiety, and affective disorder. Perlick and Russinova adopt non-HOP intervention. The former study population was caregivers of people with severe mental illness, the latter are mentally ill. It can be seen that the demographic characteristics and experience of different research participants are different, and their acceptance and focus may be different. Coupled with inconsistent focus and time of intervention, the change in this dimension of withdrawal is not significant. Further research is needed to determine the results.

All the interventions included in this review were structured randomized interventions with a model of group peer support. The quality of the included studies was high. However, there were some limitations in this study. Five of the 7 included studies used HOP interventions, which might bring publication bias. The difference between the intervention group and the control group was not simply whether there was peer support. The evidence of this review was inferred from the study results but not direct empirical evidence. The indicators measuring each outcome were not completely consistent, which resulted in poor standardization. The number of the studies reporting certain outcomes was not enough to conduct heterogeneity analyses. This meta-analysis did not include follow-up data. There was limited evidence on the long-term effectiveness of the intervention, and whether the intervention results changed over time was not demonstrated. Follow-up research needs to focus on how to maintain the effect in weeks and months after the anti-stigma intervention is completed.

Peer support is multifaceted, including social and emotional support and how to solve problems. Therefore, interventions on stigma need to be designed to match the characteristics of stigma in patients with different mental illnesses. In addition, interventions to improve stigma may be more valuable in the early phase of the disease (68). It remains a challenge to help people with mental illness to identify the symptoms, seek help, and successfully manage distressing psychiatric symptoms as early as possible through peer support to maintain daily function and improve quality of life. Peer intervention is based on the principle of homogeneity. Experiences of staying with “atypical” group members not only leads to a failure in eliminating stigma, but instead makes stereotypes more extreme (69). Whether groups can tolerate patients diagnosed with different mental illness still needs further research. For peer supporters, they have to undertake additional obligation of employing their distressing experiences. Managers need to pay more attention to the level of health and rehabilitation of peer workers so that they can be competent to work and avoid the possibility of adverse health problems. How to determine the terms of reference and responsibilities of their roles, making preparation and training in advance, etc., all require attention.

## Conclusion

Self-stigma and stigma pressure could be ameliorated by peer-led intervention, and recovery and empowerment also tended to be improved. Peer-led intervention could increase self-efficacy and the willingness to seek professional help, but had no significant effect on clinical symptoms and withdrawal. HOP intervention had significantly positive effect on disclosure-related confidentiality and distress. This review included 5 studies reporting HOP intervention, which might result in publication bias. The number of studies on some certain outcomes was not enough, and the results should be interpreted with caution.

## Data Availability Statement

The original contributions presented in the study are included in the article/supplementary material, further inquiries can be directed to the corresponding authors.

## Author Contributions

JS and HS: conception and design. HS: administrative support. JS, XY, CL, and WL: provision of study materials or patients and collection and assembly of data. JS and XY: data analysis and interpretation. All authors manuscript writing and final approval of manuscript.

## Conflict of Interest

The authors declare that the research was conducted in the absence of any commercial or financial relationships that could be construed as a potential conflict of interest.

## Publisher's Note

All claims expressed in this article are solely those of the authors and do not necessarily represent those of their affiliated organizations, or those of the publisher, the editors and the reviewers. Any product that may be evaluated in this article, or claim that may be made by its manufacturer, is not guaranteed or endorsed by the publisher.
